# Supplementary material for: Ultra-flat and long-lived plasmons in a strongly correlated oxide
Source: Nat Commun. 2022 Aug 9;13:4662. doi: 10.1038/s41467-022-32359-0 (PMC9363501; doi:10.1038/s41467-022-32359-0)
Supplement: Supplementary file 1 — Supplementary Information [file 41467_2022_32359_MOESM1_ESM.pdf]

Supplementary Information

Ultra-flat and long-lived plasmons in a strongly correlated oxide

Gao *et al*.

This document includes:

Supplementary Figures;

Supplementary Tables;

Supplementary Notes;

Supplementary References;

Supplementary Figures

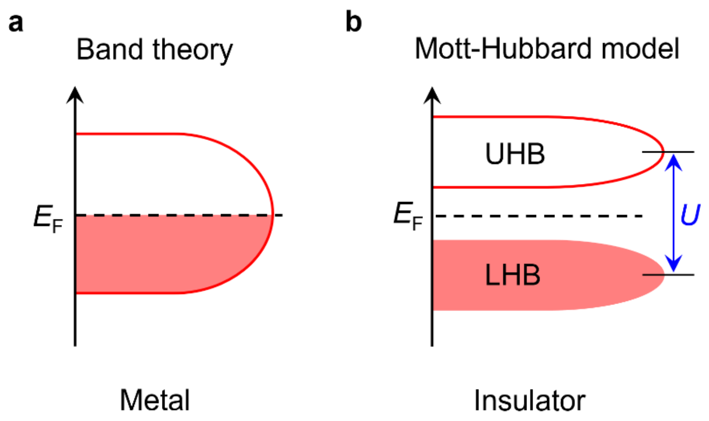

Supplementary Figure 1 | Band structures schematics for correlated systems: a, Conventional band theory and **b**, The Mott-Hubbard model.

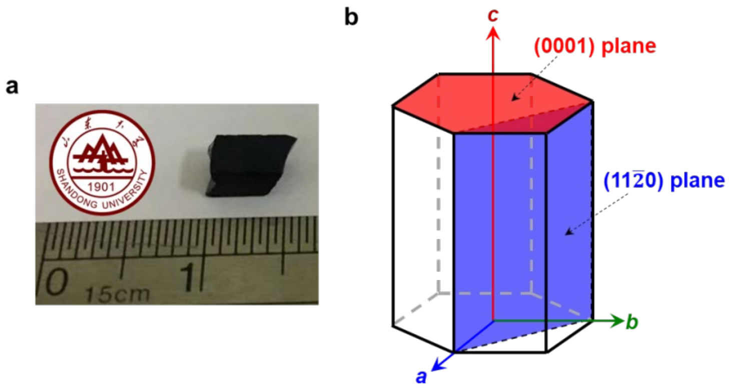

Supplementary Figure 2 | Details for the α-Ti_2O_3 single crystal. **a**, Photograph. **b**, Presentation of the (0001) and (11$\bar{2}$0) planes.

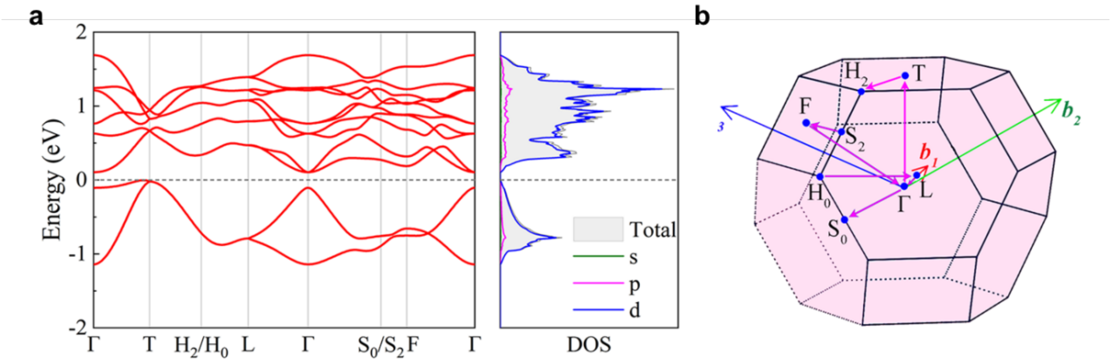

Supplementary Figure 3 │ Calculated electronic band structure for α-Ti_2O_3. a, The band structure and orbital projected density of states (DOS) for α-Ti_2O_3. The energy of the Fermi level is set to zero. **b**, Brillouin Zone with the high-symmetry *K* points and band paths.

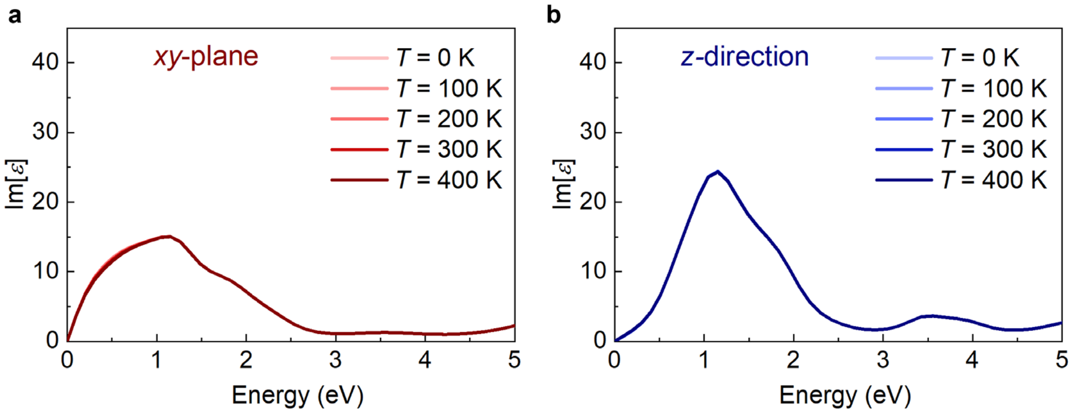

Supplementary Figure 4 | Temperature-effect on the theoretical Im(ε). Calculated imaginary part of permittivity for **a**, *xy*-plane and **b**, *z*-direction at different temperatures.

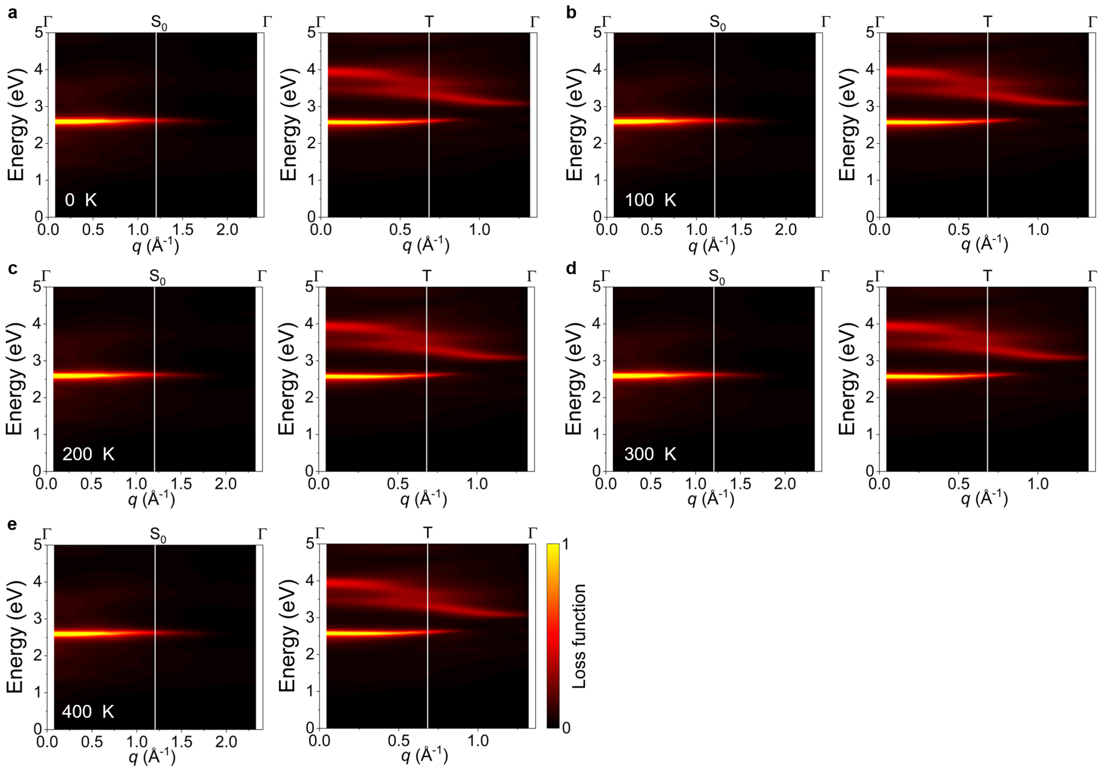

Supplementary Figure 5 | Temperature-effect on the flat plasmons in α-Ti_2O_3. Calculated EELS with LFE for α-Ti_2O_3 along Γ-S_0 and Γ-T directions at **a,** 0 K; **b,** 100 K; **c,** 200 K; **d,** 300 K; and **e,** 400 K.

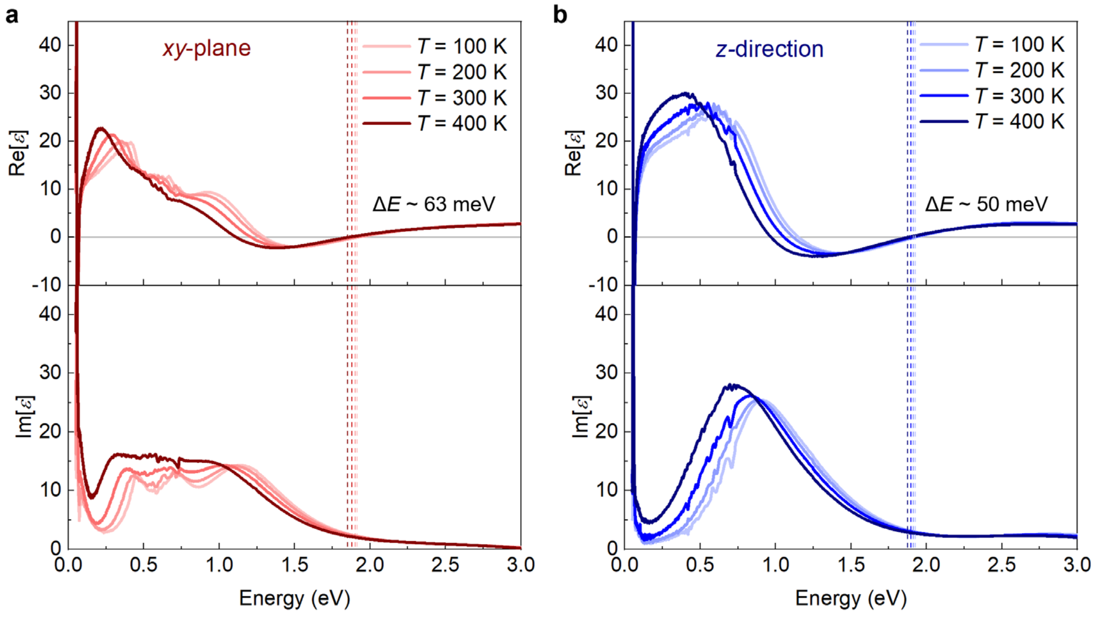

Supplementary Figure 6 | Temperature-effect on the experimental Re(ε) and Im(ε). Experimental real (upper) and imaginary part (lower) of the permittivity for **a**, *xy*-plane and **b**, *z*-direction, respectively.

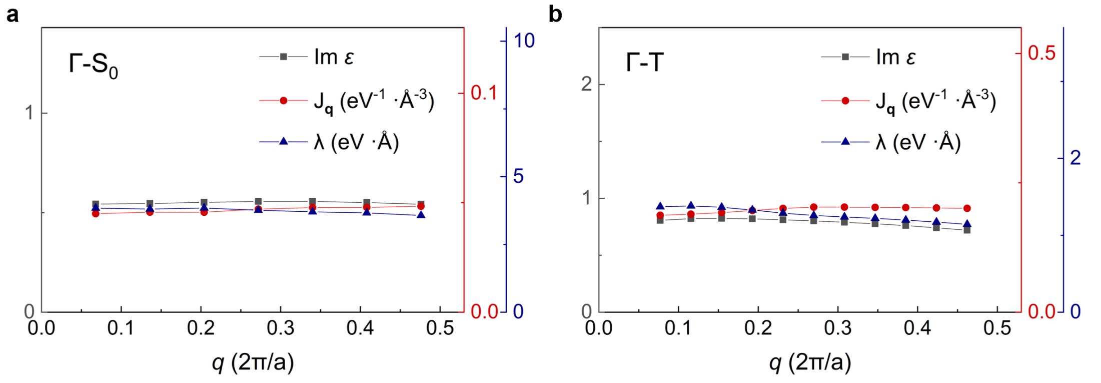

Supplementary Figure 7 | Parameters beyond the long-wavelength limit. The calculated $\mathrm{Im}[\varepsilon]$, J_q, and λ ($v(\boldsymbol{q})F(\boldsymbol{q},\hbar\omega)$) as the functions of q at the plasmon energy along **a**, Γ-S_0 and **b**, Γ-T directions, respectively.

Supplementary Tables

Supplementary Table 1. The transition dipole moments (TDMs) between electronic states at Γ . The total TDM ($\left|\langle\psi_m|\boldsymbol{r}|\psi_n\rangle\right|^2$) and its components (X: $\left|\langle\psi_m|x|\psi_n\rangle\right|^2$, Y: $\left|\langle\psi_m|y|\psi_n\rangle\right|^2$ and Z: $\left|\langle\psi_m|z|\psi_n\rangle\right|^2$) are shown for each transition process, respectively. + and − represent the parity of the electronic states. Transition between states of same parity is forbidden, as discussed in the main text.

TDM(Debye^2) / Transition	X	Y	Z	Total
1(+)→3(+)	0	0	0	0
2(−)→3(+)	26.495	112.266	1764.015	1902.776
1(+)→4(+)	0	0	0	0
2(−)→4(+)	274.223	2088.505	138.003	2500.731
1(+)→5(+)	0	0	0	0
2(−)→5(+)	0.269	0.545	1.501	2.315
1(+)→6(+)	0	0	0	0
2(−)→6(+)	0.22	3.041	1.598	4.859
1(+)→7(−)	0.587	4.287	0.236	5.111
2(−)→7(−)	0	0	0	0
1(+)→8(−)	0.045	0.341	3.764	4.150
2(−)→8(−)	0	0	0	0
1(+)→9(−)	12.153	0.006	1.916	14.076
2(−)→9(−)	0	0	0	0
1(+)→10(−)	2.309	19.212	2.091	23.612
2(−)→10(−)	0	0	0	0
1(+)→11(−)	0.387	0.523	14.966	15.876
2(−)→11(−)	0	0	0	0
1(+)→12(+)	0	0	0	0
2(−)→12(+)	55.149	0.028	8.695	63.873

Supplementary Table 2. The start/end momentum, q_{min} (Å^{-1})/$q_{\max}$ (Å^{-1}), plasmon energy, ω_p (eV), flatness, $\Delta\omega_p$ (eV), and spectral region for the flat plasmon[18,50-57] shown in Figure 5.

	Materials	q_{min} (Å^{-1})	$q_{\max}$ (Å^{-1})	ω_p (eV)	$\Delta\omega_p$ (eV)	Spectrum	Ref.
3D	Ti_2O_3(x)	0	1.32	2.64	0.04	VIS	This work
	Ti_2O_3(z)	0	0.80	2.66	0.03	VIS	This work
	CaB_2(z)	0	0.52	0.51	0.10	NIR	56
Bulk layered	Ca_2N(z)	0	0.58	0.38	0.03	MIR	57
	NbS_2(x)	0	0.40	1.08	0.04	NIR	54
	$NbSe_2$(x)	0	0.72	0.81	0.10	NIR	55
	VSe_2(z)	0	0.54	0.46	0.10	MIR	55
2D	$NbSe_2$	0	0.22	0.85	0.08	NIR	55
	TaS_2	0.17	0.40	0.95	0.10	NIR	18
	Li/C	0	0.04	2.05	0.02	VIS	52
	$B_{\triangle}$(y)	0.11	0.15	2.02	0.10	VIS	53
	$MoOCl_2$	0.18	0.25	2.47	0.10	VIS	51
	BL-MoS_2	0.09	0.20	0.29	0.09	MIR	50

Supplementary Note 1

Finite-temperature effect on the damping

Finite temperature can be a source of damping of plasmons. However, in our case, the finite temperature has neglectable impact on the damping of the plasmons (~ 2.6 eV) in α-Ti_2O_3. The reason is explained as follows.

1. Theoretical calculations

It is known that the damping of plasmons is determined by the imaginary part of dielectric function $\varepsilon_{GG'}(\boldsymbol{q},\omega)$, which can be written as

$$\mathrm{Im}\,\varepsilon_{GG'}(\boldsymbol{q},\omega) = -\frac{4\pi}{|\boldsymbol{q}+\boldsymbol{G}|^2}\mathrm{Im}\,\chi^0_{G,G'}(\boldsymbol{q},\omega)$$

where $\chi^0_{G,G'}$ is the non-interacting density response function, which is defined by

$$\chi^0_{GG'}(\boldsymbol{q},\omega) = \frac{1}{\Omega}\sum_{\boldsymbol{k}}^{\mathrm{BZ}}\sum_{n,n'}\frac{f_{n\boldsymbol{k}} - f_{n'\boldsymbol{k}+\boldsymbol{q}}}{\omega + \varepsilon_{n\boldsymbol{k}} - \varepsilon_{n'\boldsymbol{k}+\boldsymbol{q}} + i\eta} \times \langle\psi_{n\boldsymbol{k}}|e^{-i(\boldsymbol{q}+\boldsymbol{G})\cdot\boldsymbol{r}}|\psi_{n'\boldsymbol{k}+\boldsymbol{q}}\rangle\Omega_{\mathrm{cell}}$$

$$\times\langle\psi_{n\boldsymbol{k}}|e^{i(\boldsymbol{q}+\boldsymbol{G}')\cdot\boldsymbol{r}'}|\psi_{n'\boldsymbol{k}+\boldsymbol{q}}\rangle\Omega_{\mathrm{cell}}.$$

The finite temperature can affect the imaginary part of dielectric function through the Fermi distribution $f_{n\boldsymbol{k}}(E)$,

$$f_{n\boldsymbol{k}}(E) = \frac{1}{1 + exp[(E - E_F)/k_B T]}$$

where E_F is the Fermi energy, k_B and T are Boltzmann constant and absolute temperature, respectively. Then, we examined the effect of finite temperature by recalculating the Im(ε) and EELS via setting the temperature values from 0 to 400 K. As shown in Supplementary Fig. 4, the Im(ε) nearly keeps unchanged with temperature increased to 400 K, indicating the damping of plasmons is nearly not influenced by the temperature. Moreover, the difference for plasmon energy, intensity and the maximum propagating wave vector at different temperatures (Supplementary Fig. 5) is very small (~ 1%). Therefore, the finite temperature has negligible effects on the damping of plasmons in α-Ti_2O_3, based on the calculations.

It should be noted that temperature can affect the plasmon behavior via affecting electron-phonon interactions and thermal excitations of electrons, which would introduce additional dissipation channels for plasmons[S1,S2]. However, these higher-order decay processes can only affect the plasmon modes that have low energy and close to the phonon energy.

2. Experimental measurements

In fact, the temperature can change the lattice constants and correlation strength in α-Ti_2O_3, which can affect the band structure near Fermi level (indicated by the temperature-dependent resistivity result (**Fig. 1d**)). However, band changing near Fermi level can only influence the dielectric function at low energy range, which cannot affect the plasmon modes much since the plasmon energy at the much higher energy (~ 2.6 eV). As shown in Supplementary Fig. 6, the plasmon energy only changed by ~ 63 meV and ~ 50 meV for in-plane and out-of-plane directions, respectively. And the Im(ε) keeps nearly vanished at the plasmon energy for all temperatures, and does not change much with temperature. Therefore, the damping of plasmons in α-Ti_2O_3 would not be changed much by finite temperature.

Hence, since the plasmon energy in α-Ti_2O_3 is much larger than the k_BT thermal energy and phonon-related coupling energy, the impact of finite temperature on the damping of plasmons can be neglected[S1,S3]. Thus, the long-lived feature of plasmons in α-Ti_2O_3 is robust for finite temperatures.

Supplementary Note 2

Beyond the long-wavelength limit

We made an approximation when we discuss the origin for the ultra-flat behavior of the plasmons in α-Ti_2O_3 using

$$\varepsilon_{00}(\boldsymbol{q},\omega) = 1 - v(\boldsymbol{q})\frac{1}{V}\sum_{\boldsymbol{k}}^{BZ}\sum_{n,n'}\frac{f_{n,\boldsymbol{k}}-f_{n',\boldsymbol{k}+\boldsymbol{q}}}{\hbar\omega+E_{n,\boldsymbol{k}}-E_{n',\boldsymbol{k}+\boldsymbol{q}}+i\eta}\left|\langle\psi_{n,\boldsymbol{k}}|e^{-i\boldsymbol{q}\cdot\boldsymbol{r}}|\psi_{n',\boldsymbol{k}+\boldsymbol{q}}\rangle\right|^2 \quad (1)$$

The approximation $\langle\psi_{n.\boldsymbol{k}}|e^{-i\boldsymbol{q}\cdot\boldsymbol{r}}|\psi_{n'.\boldsymbol{k}+\boldsymbol{q}}\rangle \approx -i\boldsymbol{q}\cdot\langle\psi_{n.\boldsymbol{k}}|\boldsymbol{r}|\psi_{n'.\boldsymbol{k}}\rangle$ is valid under the long-wavelength limit ($e^{-i\boldsymbol{q}\cdot\boldsymbol{r}} \approx 1 - i\boldsymbol{q}\cdot\boldsymbol{r}$). Hence, $\left|\langle\psi_{n.\boldsymbol{k}}|e^{-i\boldsymbol{q}\cdot\boldsymbol{r}}|\psi_{n'.\boldsymbol{k}+\boldsymbol{q}}\rangle\right|^2 \sim q^2$ is valid in the long-wavelength limit. For the three-dimensional system (α-Ti_2O_3), $v(\boldsymbol{q}) = 4\pi e^2/\varepsilon_{\mathrm{r}}q^2$. As a result, the product $v(\boldsymbol{q})\left|\langle\psi_{n,\boldsymbol{k}}|e^{-i\boldsymbol{q}\cdot\boldsymbol{r}}|\psi_{n',\boldsymbol{k}+\boldsymbol{q}}\rangle\right|^2$ is independent of q in the long-wavelength limit. Then, the flat behavior of the plasmons can be attributed to the relatively flat band $E_{n',\boldsymbol{k}}$ (conduction band).

Beyond the long-wavelength limit, $e^{-i\boldsymbol{q}\cdot\boldsymbol{r}} = 1 - i\boldsymbol{q}\cdot\boldsymbol{r} + o(q)$ using the first-order Taylor expansion. Therefore, $\langle\psi_{n.\boldsymbol{k}}|e^{-i\boldsymbol{q}\cdot\boldsymbol{r}}|\psi_{n'.\boldsymbol{k}+\boldsymbol{q}}\rangle = \langle\psi_{n.\boldsymbol{k}}|1 - i\boldsymbol{q}\cdot\boldsymbol{r} + o(q)|\psi_{n'.\boldsymbol{k}+\boldsymbol{q}}\rangle$. Since $\langle\psi_{n.\boldsymbol{k}}|\psi_{n'.\boldsymbol{k}+\boldsymbol{q}}\rangle = 0$, $\langle\psi_{n.\boldsymbol{k}}|e^{-i\boldsymbol{q}\cdot\boldsymbol{r}}|\psi_{n'.\boldsymbol{k}+\boldsymbol{q}}\rangle = -i\boldsymbol{q}\cdot\langle\psi_{n.\boldsymbol{k}}|\boldsymbol{r}|\psi_{n'.\boldsymbol{k}}\rangle + o(q)$. Then, we further studied the product $v(\boldsymbol{q})\left|\langle\psi_{n,\boldsymbol{k}}|e^{-i\boldsymbol{q}\cdot\boldsymbol{r}}|\psi_{n',\boldsymbol{k}+\boldsymbol{q}}\rangle\right|^2$ for q extend to $0.5\times 2\pi/a$ ($2\pi/a \approx 1.14$ Å^{-1}).

According to the Kramers-Kronig relation, the real part of $\varepsilon(\boldsymbol{q},\omega)$ can be evaluated from the imaginary part,

$$\mathrm{Re}\,\varepsilon(\boldsymbol{q},\omega) = 1 + \frac{2}{\pi}P\int_0^\infty \frac{\omega'\,\mathrm{Im}\,\varepsilon(\boldsymbol{q},\omega')}{\omega'^2-\omega^2}d\omega'. \quad (2)$$

Therefore,

$$\frac{\partial\,\mathrm{Re}\,\varepsilon(\boldsymbol{q},\omega)}{\partial q} = \frac{2}{\pi}P\int_0^\infty \frac{\omega'}{\omega'^2-\omega^2}\frac{\partial\,\mathrm{Im}\,\varepsilon(\boldsymbol{q},\omega')}{\partial q}d\omega'. \quad (3)$$

The plasmon mode is determined by $\mathrm{Re}\,\varepsilon(\boldsymbol{q},\omega)=0$. The flat plasmon means that the plasmon dispersion is independent of *q*. Thus, it should satisfy $\partial\,\mathrm{Re}\,\varepsilon(\boldsymbol{q},\omega)/\partial q=0$, for $\hbar\omega$ at the plasmon energy. That is, the flat plasmon requires that $\partial\,\mathrm{Im}\,\varepsilon(\boldsymbol{q},\omega)/\partial q=0$. Notably, the supplementary Eq. (3) is mostly contributed by the term for $\omega'\approx\omega$, i.e., for $\hbar\omega'$ near the energy of plasmons.

Using supplementary Eq. (1), we can obtain the interband part of $\mathrm{Im}\,\varepsilon(\boldsymbol{q},\omega)$:

$$\mathrm{Im}\,\varepsilon^{\mathrm{inter}}(\boldsymbol{q},\omega)=\frac{g}{(2\pi)^3}\pi v(\boldsymbol{q})\sum_{v,c}\int_{BZ}d^3\boldsymbol{k}\,\delta(\hbar\omega+E_{v,\boldsymbol{k}}-E_{c,\boldsymbol{k}+\boldsymbol{q}})F_{v,c}(\boldsymbol{k},\boldsymbol{q}),\qquad(4)$$

where v, c are the band indexes of valence and conductive bands, respectively. $F_{v,c}(\boldsymbol{k},\boldsymbol{q})=\left|\left\langle\psi_{v,\boldsymbol{k}}\right|e^{-i\boldsymbol{q}\cdot\boldsymbol{r}}\left|\psi_{c,\boldsymbol{k}+\boldsymbol{q}}\right\rangle\right|^2$ is the overlap of states. $\delta(x)$ is the Dirac function. Supplementary Eq. (4) can be simplified in the form:

$$\mathrm{Im}\,\varepsilon^{\mathrm{inter}}(\boldsymbol{q},\omega)=\pi J_q(\hbar\omega)v(\boldsymbol{q})F(\boldsymbol{q},\hbar\omega),\qquad(5)$$

where $J_q(\hbar\omega)$ is the joint density of states (JDOS) for finite wave vector $\boldsymbol{q}$, which is defined as

$$J_q(\hbar\omega)=\frac{g}{(2\pi)^3}\sum_{c,v}\int_{BZ}d^3\boldsymbol{k}\,\delta(E_{c,\boldsymbol{k}+\boldsymbol{q}}-E_{v,\boldsymbol{k}}-\hbar\omega),\qquad(6)$$

$F(\boldsymbol{q},\hbar\omega)$ is the average value of $\left|\left\langle\psi_{n,\boldsymbol{k}}\right|e^{-i\boldsymbol{q}\cdot\boldsymbol{r}}\left|\psi_{n',\boldsymbol{k}+\boldsymbol{q}}\right\rangle\right|^2$ between the states satisfying $E_{n',\boldsymbol{k}+\boldsymbol{q}}-E_{n,\boldsymbol{k}}=\hbar\omega$, which is defined as

$$F(\boldsymbol{q},\hbar\omega)=\frac{\sum_{v,c}\int_{BZ}d^3\boldsymbol{k}\,\delta(\hbar\omega+E_{v,\boldsymbol{k}}-E_{c,\boldsymbol{k}+\boldsymbol{q}})F_{v,c}(\boldsymbol{k},\boldsymbol{q})}{\sum_{v,c}\int_{BZ}d^3\boldsymbol{k}\,\delta(\hbar\omega+E_{v,\boldsymbol{k}}-E_{c,\boldsymbol{k}+\boldsymbol{q}})}.\qquad(7)$$

According to supplementary Eq. (5), the requirement for the flat behaviors of plasmons $\partial\,\mathrm{Im}\,\varepsilon(\boldsymbol{q},\omega)/\partial q=0$ is satisfied when $J_q(\hbar\omega)$ and $v(\boldsymbol{q})F(\boldsymbol{q},\hbar\omega)$ are both

independent of q. In Supplementary Fig. 7, we plotted the $\mathrm{Im}\,\varepsilon$, $J_q(\hbar\omega)$ and λ ($\lambda = v(\boldsymbol{q})F(\boldsymbol{q},\hbar\omega)$) as the functions of q, for $\hbar\omega$ at the energy of plasmons. Interestingly, λ is nearly independent of q, for q extend to $0.5\times 2\pi/a$ ($2\pi/a \approx 1.14$ Å^{-1}). As expected, $J_q(\hbar\omega)$ is nearly q-independent, due to the relative flat conduction bands in α-Ti_2O_3 ($E_{c,\boldsymbol{k}+\boldsymbol{q}}$ is nearly q-independent). As a result, $\mathrm{Im}\,\varepsilon$ is nearly independent of q for $\hbar\omega$ at the plasmon energy that leads to $\partial\,\mathrm{Im}\,\varepsilon(\boldsymbol{q},\omega)/\partial q = 0$, demonstrating the flat behaviors of plasmons.

Thus, beyond the long-wavelength limit, the approximation $\langle \psi_{n.\boldsymbol{k}} | e^{-i\boldsymbol{q}\cdot\boldsymbol{r}} | \psi_{n'.\boldsymbol{k}+\boldsymbol{q}} \rangle \approx -i\boldsymbol{q}\cdot\langle \psi_{n.\boldsymbol{k}} | \boldsymbol{r} | \psi_{n'.\boldsymbol{k}} \rangle$ and q-independent $v(\boldsymbol{q})\left|\langle \psi_{n,\boldsymbol{k}} | e^{-i\boldsymbol{q}\cdot\boldsymbol{r}} | \psi_{n',\boldsymbol{k}+\boldsymbol{q}} \rangle\right|^2$ are both invalid. However, we found that the $\lambda = v(\boldsymbol{q})F(\boldsymbol{q},\hbar\omega)$ (where $F(\boldsymbol{q},\hbar\omega)$ is the average value of $\left|\langle \psi_{n,\boldsymbol{k}} | e^{-i\boldsymbol{q}\cdot\boldsymbol{r}} | \psi_{n',\boldsymbol{k}+\boldsymbol{q}} \rangle\right|^2$) is independent of q, which can lead to the flat behavior of plasmons with q-independent $J_q(\hbar\omega)$ (flat conduction bands). Hence, same conclusion, that is the flat behavior of plasmons can be attributed to the relatively flat conduction band, can be made at and beyond the long-wavelength limit. It should be noted that the analysis is performed without considering the local field effect (LFE). In principle, the results in Supplementary Fig. 7 should be more q-independent with LFE in the real material system.

Supplementary References

S1. D. Novko. Dopant-Induced Plasmon Decay in Graphene. *Nano Lett.* 17, 11, 6991 (2017).

S2. A. Iurov, *et al.* Plasmon dissipation in gapped graphene open systems at finite temperature. *Phys. Rev. B* 93, 035404 (2016).

S3. S. Xue, *et al.* Observation of Nodal-Line Plasmons in ZrSiS. *Phys. Rev. Lett.* 127, 186802 (2021).
